# Screen Time and Sleep of Rural and Urban South African Preschool Children

**DOI:** 10.3390/ijerph17155449

**Published:** 2020-07-29

**Authors:** Simone A. Tomaz, Trina Hinkley, Rachel A. Jones, Estelle D. Watson, Rhian Twine, Kathleen Kahn, Shane A. Norris, Catherine E. Draper

**Affiliations:** 1 Division of Exercise Science and Sports Medicine, Department of Human Biology, University of Cape Town, Cape Town 7700, South Africa; 2 Faculty of Health Sciences and Sport, University of Stirling, Stirling FK9 4LA, Scotland, UK; 3 Institute for Physical Activity and Nutrition (IPAN), School of Exercise and Nutrition Sciences, Deakin University, Geelong 3125, Australia; think.research@outlook.com; 4 Early Start, Faculty of Social Sciences, University of Wollongong, Wollongong 2500, Australia; rachelj@uow.edu.au; 5 Centre for Exercise Science and Sports Medicine, School of Therapeutic Sciences, University of Witwatersrand, Johannesburg 2000, South Africa; estelle.watson@wits.ac.za; 6 MRC/Wits Rural Public Health and Health Transitions Research Unit (Agincourt), School of Public Health, Faculty of Health Sciences, University of the Witwatersrand, Johannesburg 2000, South Africa; rhian.twine@wits.ac.za (R.T.); Kathleen.Kahn@wits.ac.za (K.K.); 7 South African MRC Developmental Pathways for Health Research Unit, School of Clinical Medicine, Faculty of Health Sciences, University of the Witwatersrand, Johannesburg 2000, South Africa; Shane.Norris@wits.ac.za

**Keywords:** movement behavior, pediatrics, sedentary, sitting, physical activity

## Abstract

This study aimed to investigate the extent to which preschool children meet guidelines for screen time (<1 h/day) and sleep (10–13 h/24-h) and explored home factors that affect these behaviors. Parents of preschoolers across income settings in South Africa (urban high-income *n* = 27, urban low-income *n* = 96 and rural low-income *n* = 142) completed a questionnaire. Urban high-income children had higher rates of exceeding screen time guidelines (67.0%) than children from urban low-income (26.0%) and rural low-income (3.5%) settings. Most children (81.0%) met sleep guidelines on weekdays and on weekends (75.0%). More urban high-income children met the sleep guideline, in comparison to both low-income settings. Fewer urban high-income parents (50.0%) thought that screen time would not affect their preschooler’s health, compared to urban low-income (90.4%) and rural low-income (81.7%) parents. Weeknight bedtime was positively correlated with both weekday screen time (*p* = 0.001) and weekday TV time (*p* = 0.005), indicating that more time on screens correlated with later bedtimes. Meeting screen time and sleep guidelines differs across income settings, but it is evident that parents of preschoolers across all income settings would benefit from greater awareness about guidelines.

## 1. Introduction

There has been a recent shift towards integrated 24-h movement guidelines for physical activity (PA), sedentary behavior, and sleep in preschool children. Newly released South African 24-h movement guidelines for birth to five years [1] recommend: (i) at least 180 min of PA, which should include at least 60 min of energetic play; (ii) less than 1 h of sedentary screen time; and (iii) 10 to 13 h of sleep. These movement behavior guidelines align with those released by the WHO [2], screen time guidelines released by the American Academy of Pediatrics [3], and the National Sleep Foundation guidelines [4]. Recent research from high-income countries shows that complying with these guidelines is associated with better health and developmental outcomes in preschool children [5,6]. In this age group, more time on screens has also been associated with less sleep [7].

Parents of preschool children play a pivotal role in managing their child’s behaviors, including their PA, sleep, and particularly their screen time [8]. Specifically, setting rules around screen time is important for helping young children to meet screen-time guidelines [9]. Parents’ perceptions about sleep [10] and parenting self-efficacy [11] relating to their child’s sleep are further considerations within the home environment (positive perceptions and higher parenting self-efficacy are related to longer sleep). Factors such as ethnic variation, income differences, as well as differences between urban and rural settings can impact parenting behaviors related to sleep [12,13].

Limited research has been conducted on sedentary behavior and sleep of preschool children in South Africa (SA). One study found that children from a range of income settings spend a significant portion (±73%) of their preschool day sedentary [14]. Another study with preschoolers from a low-income urban setting reported low nocturnal sleep (objectively measured) average of 9.28 h ± 0.80 per night) and particularly late bed times on average (9:36 p.m. ± 52 min) [15]. No research has addressed the extent to which preschool children in SA meet screen time and sleep guidelines, or how these behaviors relate. Factors within the home that affect preschool children’s screen time have also not been investigated across income and urban/rural settings in SA. Differences between these settings are important to consider in light of the socio-political history of SA, which significantly contributes to the current state of income inequality. Therefore, this study of preschool SA children aimed to (1) report on compliance with parent-reported screen time and sleep guidelines; (2) describe parent-reported home factors pertaining to screen time and sleep; and (3) identify differences in these outcomes between urban high-income (UH) and low-income (UL), and rural low-income (RL) settings.

## 2. Materials and Methods

Data for this study was collected in 2014–2015 as part of a larger descriptive, cross-sectional study involving preschoolers and their parents/caregivers across a range of settings in SA. Data were collected in four settings: a RL, two UL, and an UH setting, three of which have been described previously [16]. Two settings (one low- and one high-income) were based in Cape Town. The low-income setting in Cape Town was a ‘’township’’, and common challenges in this community include overcrowding, crime, unemployment, alcohol misuse, and human immunodeficiency virus/acquired immune deficiency syndrome. The second low-income urban setting was Soweto, a large urban area lining the mining belt in Johannesburg, with a mixture of low-income informal housing, as well as a rising middle class [17]. The high-income setting was a collection of suburbs in Cape Town, where the population density is approximately 15 times lower than the low-income Cape Town setting. The area has a number of high-quality private and public schools, various private health facilities and services, public parks and green spaces, expensive retailers, and well-serviced amenities. The low-income rural site was the Bushbuckridge subdistrict in Mpumalanga province, where infrastructure and the level of education are poor. Unemployment is widespread, with an estimated 60% of men and increasing numbers of women migrating to more urban areas for work [18].

**Figure 1:**
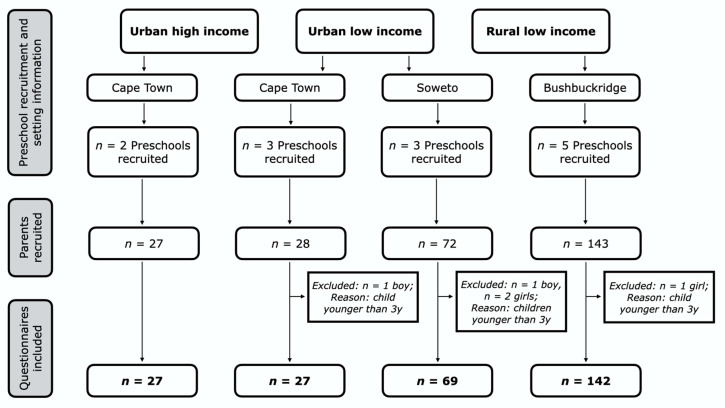
Recruitment details and exclusion for each setting.

The preschools invited to participate were intentionally diverse to ensure that they were as representative as possible, taking into account geographical location and socioeconomic status at a community level (as previously described [16]). Parents whose children met the age criteria were recruited through the preschools and contacted/invited by the relevant preschool teachers by means of parent-teacher meetings (RL and UL preschools) and preschool newsletters (UH preschools), based on the preference of the preschools. Where the parent-teacher meetings took place (at the RL and UL preschools), the parents provided consent. In these settings, fieldworkers were employed to follow up and administer the questionnaire to parents in their homes. In the UH setting, questionnaires and consent forms were dropped off at the preschools by the researcher and sent home with the children, as per the requests of the preschool teachers. The only inclusion criterion was that the child of the recruited parent needed to be in the preschool ‘’class’’, i.e., at least three years old and not in Grade 1 (the first year of formal schooling). As such, five parents/caregivers of children aged below 3 years returned questionnaires and these were excluded (see Figure 1). The sample consisted of a total of 265 parents/caregivers (hereafter referred to as parents) of preschool children (3–6 years): 27 UH parents, 96 parents from the UL settings, and 142 parents from the RL setting.

The questionnaire used to assess preschool children’s screen time and sleep, and home factors influencing preschool children’s screen time and sleep, was adapted from components of the Healthy Active Preschool Years (HAPPY) parent questionnaire [19] and the Preschool-age Children’s Physical Activity Questionnaire (Pre-PAQ) [20]. These questionnaires have been tested for reliability and validity in Australian preschoolers from different income settings [19,20]. The adapted questionnaire included questions pertaining to family demographics, screen time and sleep times, parental barriers, self-efficacy, beliefs and behaviors relating to screen time, and the use/number of screens in the home.

All analyses are stratified by setting, as it was deemed appropriate despite the vast differences in sample size, and due to differences shown between questionnaire respondents, child characteristics, as well as the main variables of interest. Analyses were not stratified by parent sex, as the majority of parents were female. Two questions that required parents to report confidence on a 3-point scale from ‘‘not at all confident’’ to ‘‘extremely confident’’ are dichotomized, with the ‘‘not at all confident’’ scores reported. Three questions that required parents to report agreement on a 5-point scale from ‘‘strongly disagree’’ to ‘‘strongly agree’’ are dichotomized as ‘‘agree’’ (‘‘strongly agree’’ and ‘‘agree’’) and ‘‘disagree’’ (‘‘strongly disagree’’, ‘‘disagree’’, and ‘‘neither agree/disagree’’) [21], with the ‘‘agree’’ scores reported. Two questions required parents to answer on the 5-point scale described as 1 = Never; 2 = Rarely; 3 = Sometimes; 4 = A lot or most of the time; and 5 = Always; the scores for ‘‘Never’’ and ‘’‘Rarely’ were combined, and scores for ‘‘A lot or most of the time’’ and ‘‘Always’’ were combined, therefore reducing the 5-point scale to a 3-point scale with all 3 scales reported (scores for ‘‘sometimes’’ remained unchanged). Statements in the questionnaire that require the parents to recall frequency of activities are reported independently.

Stata 13 for Mac (StataCorp, College Station, TX, USA) was used to perform all statistical analyses. Missing data from individual variables were excluded from analyses. Results are presented as frequencies and percentages, with sample sizes for variables stated throughout the tabulated results to indicate where data are missing. Chi-squared analyses were used to determine the differences between settings for categorical data. To determine differences between settings for continuous data, analysis of variance (ANOVA) analyses were used where data were normally distributed; for data not normally distributed, Kruskal–Wallis analyses were used. The Brown–Forsythe robust test was applied to determine significance given the unequal sample sizes. Where differences across settings were significant (after the Brown–Forsythe robust test was applied), Bonferroni and Mann–Whitney U post-hoc tests were used to determine specific between-setting differences. Spearman’s correlations were used to explore the relationship between sleep and screen time.

Ethical approval for this research was obtained from the University of Cape Town Human Research Ethics Committee (HREC REF 237/2012), the University of the Witwatersrand Human Research Ethics Committee (Medical) (M140250), and the Mpumalanga Provincial Departments of Health and Education. All parents provided written informed consent for their participation. This study adheres to the guidelines described in the Declaration of Helsinki Ethical Principles for Medical Research Involving Human Subjects [22].

## 3. Results

### 3.1. Demographic Characteristics

Table 1 presents demographic characteristics of the sample. UH children were significantly older than children from low-income settings, and parents from the UH setting were significantly older than UL parents (both *p* < 0.05). The UH and RL parents were of a similar age, even though 30% of the RL sample were grandmothers, where none of the respondents in the UH sample were grandparents. With regards to socioeconomic indicators, education levels were significantly lower amongst the low-income parents, with the RL parents having the lowest education levels (*p* < 0.05). Car ownership was significantly different between settings, with the majority of UH parents having a car, compared to less than a third of low-income parents (*p* < 0.05). The majority of parents reported owning a television, although significantly fewer parents reported this in the RL setting (*p* < 0.05).

### 3.2. Children’s Behaviors and Home Factors

Parent-reported screen time and ownership of screen-based devices are presented in Table 2. Children from UH settings have more screens and engage in more screen time, with RL children engaging the least (*p* < 0.05). In terms of overall screen time compliance, significant differences between settings were evident (Χ^2^ = 88.04, *p* < 0.001).

Table 3 reports on parents’ perceptions of their child’s screen time. The majority of low-income parents did not think that their child’s television time would affect their health, and only half of the UH parents thought that their child’s television time would affect their health. Parents from the RL setting were the least likely to limit their child’s screen time and were the least confident to influence their child’s screen time behavior. Parents’ reporting of sleep is presented in Table 4. Children in the UL setting get to bed later during the week than children in the UH and RL settings, and later on weekends than children in the RL setting. Children in the RL setting wake up earlier than the children from both urban settings on weekdays. On weekends, children from the UH setting wake up earlier than children from the low-income settings; children from the UL settings wake up later than children from the RL setting. Children from the UH setting get less total sleep than children from the low-income settings on weekends, with children from the UL setting getting the most sleep on weekends. However, this can be accounted for by the fact that more children from the low-income settings are napping during the day. In terms of meeting the guideline for 10–13 h of sleep (per 24 h), more children from the UH setting met the guideline, in comparison to the low-income settings where it was observed that 19.6% of children in the UL setting did not meet the guideline on weekdays, but 36.8% exceeded the guideline on weekends. With reference to average sleep (for week and weekend days combined), 81.6% of the children met the sleep guideline.

**Figure 2:**
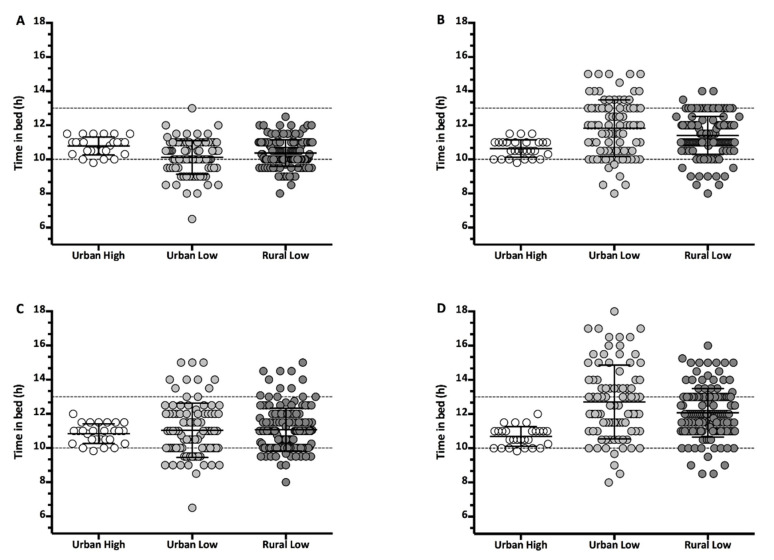
Nocturnal sleep for weekdays (**A**) and weekends (**B**), and total sleep for weekdays (**C**) and weekends (**D**), by setting.

Figure 2 shows nocturnal and total sleep for weekdays and weekend days for each setting, indicating that children in the UL setting are napping more during the day to make up for a shorter nocturnal sleep duration.

Time spent sleeping was not correlated with any screen time variables (all *p* > 0.05). However, weeknight bedtime was positively and significantly correlated with both weekday screen time (*ρ* = 0.227, *p* = 0.001, *n* = 203) and weekday TV time (*ρ* = 0.199, *p* = 0.005, *n* = 197), indicating that more time on screens correlated with later bedtimes. Weekend bedtime was not significantly correlated with weekend screen time (*ρ* = 0.140, *p* = 0.072, *n* = 166), nor weekend TV time (*ρ* = 0.128, *p* = 0.105, *n* = 162).

## 4. Discussion

While previous research shows that SA preschool children are likely to be meeting PA guidelines for their age group [16,23], these study findings reveal that fewer children are meeting guidelines for screen time and sleep. Excess screen time was the most concerning for UH children, which is contrary to research from high-income countries that has found that excess screen time amongst young children is more of a concern in low-income settings [24,25]. However, Australian preschool children from a range of income settings were also found to have low levels (17.3%) of compliance with current screen time guidelines [26].

Given that children in the UH setting had more access to screens, their excess screen time is not surprising. However, it contrasts with UH parents reporting that they limit screen time, and with them expressing high levels of confidence to manage this behavior. UH parents’ relatively low level of awareness (50%) of the impact of television on their child’s health is concerning, particularly given their higher level of education and access to information. Within this group, there appears to be a misalignment between parents’ behaviors, knowledge, and self-efficacy, and their children’s behavior, which suggests a key area for intervention. Levels of awareness (regarding the impact of television on their child’s health) were even lower amongst low-income parents, which is somewhat similar to low-income settings in the USA [24]. This is particularly problematic in the UL setting, where children are exceeding screen time guidelines on weekends, and given that in this sample, increased screen time was correlated with later bedtimes. These findings highlight the importance of disseminating information about screen time guidelines to parents of preschool children.

The proportion of children meeting sleep guidelines (81.6%) is similar to what has been found in this age group in other settings, including Australia (88.7%) [26] and Canada (83.9%) [27]. However, the between-setting differences are of interest. UH children in this study slept less on the weekends, and it is possible that this could be related to screen time on weekends, although research with larger samples is required to confirm this. The differences in nocturnal sleep between settings could potentially be explained by differences in sleep conditions and bedtime routines. Bed- and room-sharing is typical in some low-income settings [10], and bedtime routines have been found to be less prevalent in low-income settings [12].

Excess sleep amongst some UL children also warrants further investigation to see if this is replicated in larger samples from these types of settings. Research with adults and older adults in similar UL settings in SA shows a tendency for longer sleep duration [28,29]. The findings presented in this paper suggest that this sleep pattern might start at a young age in these settings, or that the habit of prolonged sleep amongst adults in a household could be passed on to children. Since excess sleep may be detrimental to the health and well-being of school-aged [30] and younger children [6], future research and intervention strategies with preschool children should take both insufficient and excess sleep into account.

The findings from the rural parents are perhaps the most interesting. Although children in this setting have the least access to screens and reportedly engage in the least screen time, rural parents were the least likely to have rules about screen time and were the least confident to influence their child’s screen time behavior. This may be because in these settings there is less need for rules pertaining to screen time. However, considering the rapidly transitioning nature of settings such as these in SA [31] and thus the potential increase in access to screens, it would be worth tracking these behaviors into later childhood, as screen time and sleep may not remain in the healthy range.

In terms of factors within the home that could be associated with screen time and sleep behaviors in this study, this study only examined a limited number of factors within the home and did not fully explore the complexity of the home environment in terms of its potential influence on health behaviors. However, the findings of this study suggest that the impact of household-level income disparities on preschool children’s health behaviors need to be investigated further. This may include exploring the impact of the individual playing the primary caregiving role and the age of this caregiver (e.g., older caregivers such as grandparents have been found to perceive screen time negatively and so are inclined to limit it in low-income settings [32]). The predominance of female caregivers in the sample is not unexpected since many children in low-income SA settings grow up without a father in the household [33], but contextual differences need to be taken into consideration when intervening to promote healthy behaviors amongst young children in SA.

The main limitation of this study is the reliance on parent-report data. However, in the absence of an objective measure of screen time, self-report is the most feasible means of assessing this behavior. Another major limitation of this study is the vast difference between sample sizes for each income group. Collecting data from UH parents proved to be particularly challenging, and statistical methods were applied where appropriate to correct for the discrepancies. This study was also limited by the lack of a specific indicator to determine high- and low-income status. The settings were classified at a community level, rather than a household or individual level. Despite these limitations, it is important to note that this is the first study to assess parent-reported preschool children’s screen time and sleep behaviors in SA and to look at differences across income settings. Another limitation of the study was the use of a questionnaire not validated in SA. However, no validated questionnaire exists for this age group in SA, and so the most appropriate questionnaires were chosen and adapted. In an attempt to adapt the HAPPY and Pre-PAQ questionnaires to make the final questionnaire applicable across a wide range of settings in SA, it is possible that certain questions were not relevant in some settings. This may have led to spurious responses, especially in the low-income settings where levels of education and literacy were lower, although the assistance of a local fieldworker was intended to minimize these types of responses.

## 5. Conclusions

This study contributes to a growing body of literature on screen time and sleep in SA preschool children, and the extent to which children in this age group are meeting relevant guidelines. Although there are differences in behaviors across income settings, it is evident that parents of preschool children in all settings would benefit from greater awareness about guidelines for screen time and sleep in this age group.

## Figures and Tables

**Table 1:** Demographic characteristics of parents/caregivers by income setting.

Characteristics	Total (*n* = 265)	UH (*n* = 27)	UL (*n* = 96)	RL (*n* = 142)
Age (years)				
Caregiver (*n* = 256)	37.5 ± 11.7	41.4 ± 4.9 ^a^	34.0 ± 7.8	39.1 ± 13.9
Child (*n* = 259)	4.8 ± 0.7	5.3 ± 0.5 ^a,b^	4.7 ± 0.7	4.8 ± 0.6
Relationship to the preschool child (*n* = 263) *				
Mother	194 (73.8%)	26 (96.3%)	80 (85.1%)	88 (62.0%)
Father	11 (4.2%)	1 (3.7%)	7 (7.5%)	3 (2.1%)
Grandmother	47 (17.9%)	0	4 (4.3%)	43 (30.3%)
Other	11 (4.2%)	0	3 (3.2%)	8 (5.6%)
Highest level of education (*n* = 260) *				
Grade 9 or lower	46 (17.7%)	0	4 (4.4%)	42 (29.8%)
Grade 10–11	54 (20.8%)	0	19 (20.7%)	35 (24.8%)
Grade 12/matriculated	90 (34.6%)	4 (14.8%)	42 (45.7%)	44 (31.2%)
Tertiary diploma/certificate	39 (15.0%)	7 (25.9%)	14 (15.2%)	18 (12.8%)
University degree	31 (11.9%)	16 (59.3%)	13 (14.1%)	2 (1.4%)

Data presented as *n* (%), except for age (years). UH = urban high-income, UL = urban low-income, RL = rural low-income. ‘‘Other’’ relationship includes grandfathers, aunts, and uncles. * *p* < 0.05 for difference between groups after robust test for unequal sample size applied. ^a^ Significant difference between UH and UL; ^b^ Significant difference between UH and RL; *p* < 0.05.

**Table 2:** Parent report of availability of screen devices and child screen time by setting.

Screen Time Variables	Total	UH	UL	RL
*Smart phone/digital tablet (e.g., iPad)*	(*n* = 255)	(*n* = 27)	(*n* = 86)	(*n* = 142)
Has in the home (%) *	78.1	92.6	78.1	75.4
Engages in this screen time (*n* (%)) *	68 (25.9%)	20 (74.1%)	33 (40.7%)	11 (7.8%)
Total time: weekdays (h/wk) ^†^	1.74 ± 1.22	1.96 ± 1.63	1.65 ± 1.04	1.64 ± 1.00
Total time: weekend (h/wkd) ^†^	2.01 ± 1.65	1.45 ± 1.09	2.79 ± 1.92	0.83 ± 0.29
*TV/video’s/DVDs*	(*n* = 256)	(*n* = 27)	(*n* = 87)	(*n* = 142)
Has in the home (%)	84.9	96.3	90.6	78.9
Engages in this screen time (*n* (%))	223 (87.1%)	27 (100%)	76 (87.4%)	120 (84.5%)
Total time: weekdays (h/wk) ^†,^*	2.72 ± 2.08	5.08 ± 3.62 ^a,b^	2.90 ± 1.31 ^c^	2.11 ± 1.45
Total time: weekend (h/wkd) ^†,^*	2.71 ± 1.87	3.75 ± 2.32 ^a^	3.68 ± 2.11 ^c^	1.87 ± 1.00
*Playstation^©^/Nintendo^©^/X-Box^©^/Gameboy^©^/other computer games*	(*n* = 256)	(*n* = 27)	(*n* = 87)	(*n* = 142)
Has in the home (%)	15.8	63.0	14.6	7.8
Engages in this screen time (*n* (%)) *	22 (8.6%)	6 (22.2%)	14 (16.1%)	2 (1.4%)
Total time: weekdays (h/wk) ^†^	2.63 ± 2.17	1.55 ± 0.84	3.4 ± 2.71	2.50 ± 2.12
Total time: weekend (h/wkd) ^†,^*	2.58 ± 2.04	1.10 ± 0.73	3.86 ± 1.95 ^a^	0
*Computer/internet (excluding games)*	(*n* = 256)	(*n* = 27)	(*n* = 87)	(*n* = 142)
Has in the home (%)	26.4	88.9	29.2	12.7
Engages in this screen time (*n* (%))	14 (5.5%)	2 (7.4%)	7 (8.1%)	5 (3.5%)
Total time: weekdays (h/wk) ^†^	2.33 ± 1.60	5.0 ± 0	1.7 ± 0.97	1.9 ± 1.34
Total time: weekend (h/wkd) ^†^	2.56 ± 2.06	5.5 ± 0.7	1.8 ± 1.10	0.50 ± 0
*Children meeting the screen time guideline (<1 h/day)* * (%)	(81.9%)	(33.3%)	(74.0%)	(96.5%)
*Total screen time: weekdays (h/wk)*^†,^*	(*n* = 206) 3.38 ± 3.09	(*n* = 26) 6.91 ± 5.03 ^a,b^	(*n* = 61) 3.89 ± 2.77 ^c^	(*n* = 119) 2.35 ± 1.83
*Total screen time: weekend (h/wkd)*^†,^*	(*n* = 170) 3.44 ± 3.23	(*n* = 27) 5.33 ± 3.83 ^a,b^	(*n* = 52) 5.18 ± 3.99 ^c^	(*n* = 91) 1.88 ± 1.02

UH = urban high UL = urban low-income, RL = rural low-income. * *p* < 0.05 for difference between groups after robust test for unequal sample size applied. ^a^ Significant difference between UH and UL; ^b^ Significant difference between UH and RL; ^c^ Significant difference between UL and RL; *p* < 0.05. ^†^ Data shown only for children who engage in screen time with this device.

**Table 3:** Parent perceptions of preschool child’s screen time.

Screen Time Perceptions	Total (*n* = 264)	UH (*n* = 27)	UL (*n* = 95)	RL (*n* = 142)
*Child is more likely to watch TV than be active (%)* *				
Never/Rarely	43.6	11.1	15.8	68.3
Sometimes	36.7	77.8	43.2	24.7
Most of the time/Always	18.9	7.4	41.1	6.3
*Child is more likely to play electronic games than be active (%)* *				
Never/Rarely	59.5	44.4	32.3	80.3
Sometimes	19.9	37.0	21.5	15.5
Most of the time/Always	19.1	11.1	44.1	4.2
Parent believes amount of TV child watches would not affect his/her health (%) ^†,^*	81.7	50.0	90.4	81.7
Parent limits TV time (%) ^†,^*	74.8	76.9	90.1	64.5
Parent limits computer and electronic game time (%) ^†,^*	68.9	81.5	87.5	54.9
Parent not confident to get child to choose activity instead of computer/e-games (%) ^β,^*	33.3	3.7	10.9	53.5
Parent not confident to say no to child’s requests to play on computer/e-games (%) ^β,^*	42.4	7.4	14.8	66.2

Data presented as %. ^†^ = % who agree. ^β^ = % not at all confident. UH = urban high-income, UL = urban low-income, RL = rural low-income. * *p* < 0.05 for difference between groups.

**Table 4:** Parent report of child sleep behaviors on weekdays and weekend days, by setting.

Sleep Variables	Total	UH	UL	RL
Bedtime: weekdays (h:mm) *	(*n* = 262) 19:55 ± 00:47	(*n* = 27) 19:45 ± 00:31	(*n* = 93) 20:23 ± 00:53 ^a,c^	(*n* = 142) 19:39 ± 00:37
Wakeup time: weekdays (h:mm) *	(*n* = 261) 06:15 ± 00:39	(*n* = 27) 06:32 ± 00:30 ^b^	(*n* = 92) 06:32 ± 00:38 ^c^	(*n* = 142) 06:01 ± 00:35
Bedtime: weekends (h:mm) *	(*n* = 257) 20:06 ± 00:55	(*n* = 27) 20:07 ± 00:43	(*n* = 89) 20:20 ± 01:10 ^c^	(*n* = 141) 19:57 ± 00:43
Wakeup time: weekends (h:mm) *	(*n* = 258) 07:34 ± 01:10	(*n* = 27) 06:46 ± 00:41 ^a,b^	(*n* = 89) 08:10 ± 01:18 ^c^	(*n* = 142) 07:22 ± 00:59
Total sleep per 24-h: weekdays (h)	(*n* = 261) 11.04 ± 1.33	(*n* = 27) 10.84 ± 0.57	(*n* = 92) 11.04 ± 1.59	(*n* = 142) 11.08 ± 1.25
Total sleep per 24-h: weekends (h) *	(*n* = 255) 12.14 ± 1.75	(*n* = 27) 10.69 ± 0.57 ^a,b^	(*n* = 87) 12.71 ± 2.16 ^c^	(*n* = 141) 12.07 ± 1.42
Children nap during the day (%) *	(*n* = 261) 53.6	(*n* = 27) 14.8	(*n* = 92) 71.7	(*n* = 142) 49.3
Nap duration: weekdays (h)	(*n* = 117) 1.63 ± 0.88	(*n* = 1) 1.5 ± 0	(*n* = 49) 1.80 ± 1.00	(*n* = 67) 1.50 ± 0.76
Nap duration: weekends (h)	(*n* = 110) 1.68 ± 0.93	(*n* = 1) 1.5 ± 0	(*n* = 44) 2.01 ± 1.07	(*n* = 65) 1.45 ± 0.75
*Sleep guideline adherence: weekdays (%)* *	(*n* = 261)	(*n* = 27)	(*n* = 92)	(*n* = 142)
Insufficient sleep	12.3	3.7	19.6	9.2
Sufficient sleep	80.8	96.3	70.7	84.5
Excess sleep	6.9	0	9.8	6.3
*Sleep guideline adherence: weekends (%)* *	(*n* = 255)	(*n* = 27)	(*n* = 87)	(*n* = 141)
Insufficient sleep	3.9	3.7	4.6	3.6
Sufficient sleep	74.9	96.3	58.6	80.9
Excess sleep	21.2	0	36.8	15.6

UH = urban high-income, UL = urban low-income, RL = rural low-income. * *p* < 0.05 for difference between groups. ^a^ Significant difference between UH and UL; ^b^ Significant difference between UH and RL; ^c^ Significant difference between UL and RL; *p* < 0.05.
